# Editorial: Creativity: Education and Rehabilitation

**DOI:** 10.3389/fpsyg.2019.01500

**Published:** 2019-06-26

**Authors:** Massimiliano Palmiero, Laura Piccardi, Raffaella Nori, Liana Palermo, Carola Salvi, Cecilia Guariglia

**Affiliations:** ^1^Cognitive and Motor Rehabilitation and Neuroimaging Unit, IRCCS Fondazione Santa Lucia, Rome, Italy; ^2^Department of Life, Health and Environmental Sciences, University of L'Aquila, L'Aquila, Italy; ^3^Department of Psychology, University of Bologna, Bologna, Italy; ^4^Department of Medical and Surgical Sciences, University Magna Graecia, Catanzaro, Italy; ^5^Department of Psychology, Northwestern University, Evanston, IL, United States; ^6^Shirley Ryan AbilityLab, Chicago, IL, United States; ^7^Department of Psychology, University of Rome ‘Sapienza’, Rome, Italy

**Keywords:** creativity, divergent thinking, children, aging, pedagogy, representation

Creativity is a multifaceted phenomenon which entails generation of new ideas to solve problems and produce innovation. It is also conceived as a means to improve the quality of life, and promote general health in both normal and clinical populations. This has progressively lead the scientific community to investigate creativity, and its inherent components (e.g., divergent thinking), in terms of cognitive processes (Palmiero et al., 2016a,b; Zedelius and Schooler, 2016) and age-related changes (Palmiero, 2015; Palmiero et al., 2017) to support educational success (Plucker et al., 2004), active aging (Palmiero et al., 2016c), and rehabilitation (Palmiero et al., 2012).

Although creativity represents a window for expressing the self and enhancing well-being, several issues remain to be investigated to clarify the extent to which idea generation and/or art expression can be used for educational and rehabilitation purposes. This Frontiers in Cognition Research Topic was explicitly devoted to shed light to some extent on these issues. Ten novel publications were collected: 7 Original Research Articles, 1 Brief Research Report Article, and 2 Reviews.

Regarding the role of creativity in educational settings, an enriching program for gifted children (6 to 10-years old) in the areas of language and science showed positive effects only on intelligence operating in well-defined problem space (Welter et al.). In this vein, in children and adolescents, drama pedagogy training was related to divergent thinking and problem-solving if based on storytelling, pretend-play and playfulness, and to risk or perspective taking if based on improvisation and role-play (Celume et al.). Interestingly, multiple sessions of improvisational theater improved both teenagers' flexibility and originality of verbal divergent thinking (Hainselin et al.). These studies showed the potential of creativity in educational settings in a wide range of domains, suggesting the need to operate in different types of problem space. Other studies highlighted the key role of mediating factors. Children (6 to 10-years old) practicing dances showed higher ability in representing the topological map of the body, which mediated their divergent thinking ability in motor domain (Palmiero et al.). Adolescents' (16 to 19-years old) cognitive self-regulation mediated the relationships between mind wondering and verbal divergent thinking (Preiss et al.). In people aged 12–88 years, gender, age, and composite intelligence quotient predicted creative achievement, whereas interpersonal emotional competence predicted creative style (Nori et al.).

Regarding the role of creativity in rehabilitation settings, spatial distortions in patients' drawings after right brain damage (unilateral spatial neglect, hyperschematia, constructional apraxia) was related to the space representation in art (Rode et al.). This means that the understanding of the pathological mechanisms underlying these disorders provides not only an opportunity to clarify visual art, but also gives the possibility to use visual artistic creativity to improve the spatial distortions after brain damages. Moreover, verbal divergent thinking, which positively correlated to the integration of the default mode network and cerebellum, was suggested for treating depression (Feng et al.), which is characterized by a reduced functional connectivity between the cerebellum and the default mode network (Liu et al., 2012). Yet, creativity was associated with the rehabilitation of schizophrenia: high schizotypal individuals exhibited higher over-inclusive thinking and cognitive inhibition, which partially mediated the relationship between schizotypy and creativity (Wang et al.). Finally, the relationships between different proxies of cognitive reserve and verbal divergent thinking were also showed, in light of the creative jobs rather than of the job complexity (Colombo et al.), confirming that creativity, and its components, might be aimed at fostering active aging.

In summary, from the knowledge we acquired by the studies collected in this Research Topic, it is possible to underline that creativity represents a flexible tool for enhancing cognition and promoting well-being during the lifespan. Much work is still necessary, of course, but there is no doubt that the application of creativity in education and rehabilitation settings represents a new research frontier. Moving forward, the challenge will be to understand the interrelations between creativity, cognitive, extra-cognitive, and personality factors in order to foster efficient educational and rehabilitation programs. Importantly, given that creativity involves different sub-components, such as divergent and convergent thinking, it would be interesting to investigate these components more systematically in future studies. Then, it might be useful to also consider the efficacy of creativity in educational and rehabilitation settings in relation to different domains of knowledge (e.g., visual, auditory, motor, etc.).

In conclusion, the variety of approaches and insights gained certainly offer a new and encouraging window to continue to work in this fascinating field.

## Author Contributions

MP: planned the topic and edited the most of papers included in the topic. LaP: planned the topic. RN, LiP, CS, and CG: edited some papers included in the topic.

### Conflict of Interest Statement

The authors declare that the research was conducted in the absence of any commercial or financial relationships that could be construed as a potential conflict of interest.
